# Social Behaviour of Horses in Response to Vocalisations of Predators

**DOI:** 10.3390/ani10122331

**Published:** 2020-12-08

**Authors:** Iwona Janczarek, Anna Wiśniewska, Michael H. Chruszczewski, Ewelina Tkaczyk, Aleksandra Górecka-Bruzda

**Affiliations:** 1 Department of Horse Breeding and Use, Faculty of Animal Sciences and Bioeconomy, University of Life Sciences in Lublin, 20-950 Lublin, Poland; iwona.janczarek@up.lublin.pl (I.J.); anna.wisniewska@up.lublin.pl (A.W.); ewelina.tkaczyk@up.lublin.pl (E.T.); 2 Faculty of Psychology, University of Warsaw, 00-183 Warsaw, Poland; mikael@psych.uw.edu.pl; 3 Department of Animal Behaviour and Welfare, Institute of Genetics and Animal Biotechnology, Polish Academy of Sciences, Jastrzębiec, 05-552 Magdalenka, Poland

**Keywords:** horse, predator, vocalisation, social defensive behaviour, grey wolf, Arabian leopard, golden jackal

## Abstract

**Simple Summary:**

Animal social strategies are of importance when avoiding predation. Since horses are the least hunted among all farm animal species, we suppose that the alert reaction to a predator’s vocalisation, followed by anti-predator social behaviour, still exists in domestic horses. Recorded vocalisations of three different predators (grey wolf, Arabian leopard and golden jackal) were played to 20 horses of two horse breeds—namely, Konik polski and Arabian. Social responses and tactics in antipredator behaviour differed between the breeds and between predators. Koniks exposed to vocalisations of a howling wolf resulted in tight groupings, while Arabians exposed to the growling of a leopard responded with linear group formation. The behaviour of studied horses, expressed by alertness and defensive formations, indicates existence of the social anti-predator behavior, which in turn may explain the low rates of horses falling prey to predators as compared with other farm animal species.

**Abstract:**

We tested the hypothesis that social defensive responses to the vocalisation of a predator still exist in horses. The recordings of a grey wolf, an Arabian leopard and a golden jackal were played to 20 Konik polski and Arabian mares. Durations of grazing, standing still, standing alert and the number of steps in walk and trot/canter were measured. In one-minute scans, the distances of the focal horse from the reference horse (DIST-RH) and from the nearest loudspeaker (DIST-LS) were approximated. The vocalisation of a leopard aroused the Arabians more than the Koniks (less grazing, stand-still and walk, more stand-alert and trotting/cantering). Koniks showed more relaxed behaviours to the leopard vocalisation (more grazing, stand-still and walk), but high alertness to the wolf playback (stand-alert, trotting/cantering). Spatial formation of the herd of Koniks showed tight grouping (lower DIST-RH) and maintaining distance from the potential threat (DIST-LS) in response to the wolf howling, while the Arabians approached the loudspeakers in linear herd formation when the leopard growls were played. Adult horses responded to potential predation by changing spatial group formations. This ability to apply a social strategy may be one of the explanations for the least number of horses among all hunted farm animal species.

## 1. Introduction

Farm animals have been predated by large carnivores from the beginning of domestication, e.g., [1,2,3,4]. The damages to livestock caused by predation [5,6], as well as the cultural and public fear of predators [7,8,9] has resulted in the almost total extermination of the majority of large predators [10]. This applies to the grey wolf (*Canis lupus*) in North America and Europe, jaguars (*Panthera onca*) in Central and South America, Arabian leopards (*P. pardus nimr*), African wild dogs (*Lycaon pictus*) in Africa and tigers (*P. tigris*) in Asia [7,11]. In Europe, four big carnivore species, the brown bear (*Ursus arctor*), the grey wolf (*Canis lupus*), the Eurasian lynx (*Lynx lynx*) and the wolverine (*Gulo gulo*), were almost driven to extinction in the last century. However, the populations of certain predator species, such as golden jackal (*Canis aureus*) in Africa and Asia [12], remain at a stable level. It has been observed that due to recent snowless winters, this species has been able to colonise new habitats in Europe [13,14].

The protection programmes introduced in America and Europe enabled saving of the last wild wolves, bears and lynxes by providing compensations to farmers for killed livestock [9,15]. With a confirmed increase in the population of wolves, the problem with depredation has recently recurred [16].

According to [17], to an extent wild prey animals are able to counteract predation. For example, bold bighorn ewes (*Ovis Canadensis*) demonstrated low rates of being preyed upon [18], while male roe deer (*Capreolus capreolus*), who are more active than females, more successfully avoided predation from red foxes (*Vulpes vulpes)* [19]. Considering the high levels of vulnerability of domestic animals to depredation, it has been suggested that the decrease in the ability to counteract the depredation is likely due to the selection for tameness in cattle [20,21], sheep [22,23] and horses [24,25]. Leopards were observed to prey also on small domestic animals [4,26]. Although it was proposed that the predispositions (including tameness) to being domesticated are embedded in their nature [27], it is clear that, before domestication, these animals were able to avoid predation sufficiently to assure the survival of the species in natural habitats.

Among all farm animals, horses are the least predated upon species [9]. It is not known if this is related to the overall smaller number of horses compared with other farm species or to their specific behaviour in response to predators. Horses are a highly vigilant species and easily express alert and/or flight behaviour, as confirmed by all studies on equine temperament and personality [28]. The tendency to react with fear is deeply rooted in the equine personality and very difficult, if even possible, to eradicate. The fearfulness, proposed as one of the personality traits in horses [24,29,30] and other animals [31] has been explained as basic, genetically imprinted response enabling threat avoidance [29]. Although this characteristic can cause troubles for humans, it has functional significance to horses as it allows them to avoid predation. The purported predator-related response has been used to exemplify equine avoidance of humans in so-called “natural” training methods [32]. Although the simple translation of horse anti-predatory behaviour into an avoidance response to humans was criticised [33], it is striking that only four reports on horse reactions to predator cues could be found in the scientific literature [24,25,34,35].

The fear response of prey animals to predators involves the processing of complex cues involving visual, olfactory and auditory stimuli. The studies on the behavioural and physiological (activation of the hypothalamic–pituitary–adrenal axis) responses to separate olfactory and auditory predator cues showed that they still evoke the instinctive state of fear in domestic horses [24,25]. In the cited studies, only the responses of individually tested horses were examined. However, most of the farm animal species are social; thus, instinctive social behaviour against predators may be of importance when avoiding predation [20,36]. It was recently confirmed by our studies [34,35] that the heart rate of horses increases during exposure to predators vocalisation. To our knowledge, the response of equine social groups (herds) to the presence of predator cues was not previously examined scientifically except for the latter studies.

In this study, we tested the hypothesis that the social behavioural response of horses to a type of predator cue, namely the auditory stimulus, still exists in domestic horses. We chose only vocal cues, as it was observed that predators vocalise before [37] or during the chase or harassment of prey [38]. As the method of hunting differs between canids and large cats (chasing or stalking, [20], we also investigated the effect of predator species on the behaviour of horses.

## 2. Material and Methods

### 2.1. Ethical Note

The procedures were conducted with permission (27/2016 issued on 13 May 2016) of the Local Commission for Ethics in Animal Experimentation, Lublin, Poland.

### 2.2. Animals

The behaviour of horses monitored with heart rate recorder in [34] was observed. Twenty adult horses (all mares), 10 Konik polski horses (Koniks) and 10 Arabian pure breed horses (Arabians), from two different farms, located at a distance of 60 km apart (Koniks: 51°32′ N, 22°97′ E and Arabians: 50°96′ N, 23°07′ E) were tested. The horses were 5–15 years old, were clinically healthy and had not been used for riding for at least 3 years. In both farms, located far from busy roads, the horses were submitted to necessary husbandry procedures (hoof trimming, herding, deworming, etc.) and were exposed to the sounds of routine agricultural works. They were kept in 3 × 3 m boxes with straw bedding and were out on grassy paddocks always in the same, socially stable group for at least 5 h daily. The horses were fed hay and oats twice daily with water and mineral salt blocks available *ad libitum*. They were habituated to humans during daily handling. For this study, before each test, the horses were identified with non-permanent numbers marked on their hindquarters.

### 2.3. Experiments

Two large predators: the wolf (*Canis lupus*) and the Arabian leopard (*Panthera pardus nimr*) were chosen for the experiment. The vocalisation of the golden jackal (*Canis aureus*), a smaller opportunistic feeder, was chosen as the control sound. The recordings of the wolf howls (W), Arabian leopard roars (P) and jackal barking howls (J) were obtained from the archives of the Department of Zoology and Animal Ecology, the University of Life Science in Lublin. The parameters of the sound were measured before the experiments. The sound level (A-weighted equivalent continuous sound level, LAeq) of the three predator vocalisations measured at the loudspeaker ranged from 54.0 dB for the jackal to 58.6 dB for the wolf. The fast time-constant sound minimum level (LAFmin) ranged from 37.7 dB for the leopard to 43.6 dB for the wolf, whereas the LAFmax ranged from 65.7 dB for the wolf to 82.5 dB for the leopard. The maximum of the acoustic pressure was registered at 1000 Hz in all cases. The wolf howls were mainly of low frequency (250–2000 Hz), those of the jackal were dominated by higher frequencies (630–5000 Hz), whereas the leopard roars were of a broader range (250–5000 Hz).

The experiments were conducted from August to September in familiar square grassy paddocks of 0.5 ha on almost windless days (0–0.5 m/s as measured with a GM 8908 anemometer) with stable weather conditions (no rain and a mean temperature of 12.2 ± 2.9 °C). All animals were naïve to the predator vocalisations or stalking. For this study, the recordings of horses submitted to six trials (days) and uniform testing protocol were taken. On each trial, the vocalisations of predators were played from one (1LS) or four (4LS) loudspeakers, positioned at 1 m distance from one (1LS) or each (4LS) corner of the familiar pasture. The order of exposure to the predator vocalisations in all six trials was semi-random. The testing days were spaced by at least 9-day intervals.

**Figure 1:**
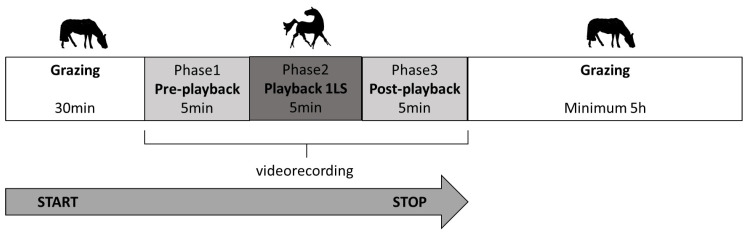
Testing protocol in each trial.

In each trial in the morning, the horses were run out together to the familiar, rectangular paddock of 0.5 h. The testing protocol was preceded by 30 min of grazing with no disturbance (Figure 1). Then, the video cameras (SONY FDR-AX33) were switched on. The cameras, the loudspeakers and the experimenter were camouflaged and not visible to the horses. After 5 min (phase 1: pre-playback), the recording with the predator vocalisation was played remotely for 5 min (phase 2: playback phase) and the subsequent 5 min were also registered (phase 3: post-playback). After the test, the horses were left grazing undisturbed for approximately 5 h.

### 2.4. Behavioural Variables

The behaviours indicating alertness and fear responses, i.e., looking around, scanning the environment, regrouping, changing the head and ear positions, beginning to move from a stationary position (walk) and engaging in energetic gaits (trot or canter) were defined and measured (Table 1) during the 5-min phases. All behaviours were assessed during the total time the horse was visible in the video-recording.

### 2.5. Distance from the Reference Horse and the LS

To measure the social reaction of the herd to the vocalisation of the predator, in each breed, the reference animal was arbitrarily indicated by the stable manager as the most dominant individual in the group. This horse served as the reference for the distance from the focal horse (DIST-RH). The video-recordings of each trial were scanned every one minute and the DIST-RH values in each of five successive scans were approximated in cm. The distances of the focal horse from the nearest loudspeaker (DIST-LS) were measured using the same method.

### 2.6. Statistical Analyses

All behavioural variables and distances were not normally distributed. For STAND-S, STAND-A and GRAZ, a binomial distribution was assumed (the duration of behaviour in proportion to the time the horse was visible). WALK, TROTCANT, DIST-RH and DIST-LS were log-transformed adding a constant value (100) to avoid 0 values when log-transforming. Then, normal distribution was tested.

The statistical models included the breed (Koniks or Arabians), the predator species (wolf, leopard or jackal), the phase (1: pre-playback, 2: playback or 3: post-playback), the number of loudspeakers (1LS or 4LS) and their interactions as classification variables. The random effect of the horse was included in all models accounting for repeated measurements taken on the same animal. The least-squares means presented in the text were calculated for each class of classification variables and compared using a Bonferroni adjustment for multiple comparisons. The level of probability was set at *p* < 0.05. The statistical analyses were made using the generalised linear mixed model analysis (GLIMMIX procedure) for STAND-S, STAND-A and GRAZ and general linear models with fixed and random effects (MIXED procedure) for WALK, TROTCANT, DIST-RH and DIST-LS (SAS statistical package, SAS 9.4., SAS Inst., Inc., Cary, NC). Additionally, the effect of playback (phase 2) from 1LS and 4LS on the number of horses grazing, standing still, standing alert, walking, trotting or cantering, dichotomized as per the median, was tested with the Chi-square test (CHISQ; IBM SPSS for Windows PS Imago 6). Only significant results are presented in result section. For better clarity of figures, the results for STAND-S, STAND-A, GRAZ, WALK and TROTCANT are shown using descriptive statistics and row data (for DIST-RH and DIST-LS).

## 3. Results

### 3.1. Behavioural Responses

All models proved that the number of loudspeakers did not affect the behavioural variables. The horses were visible on video for 68 ± 23.3% of time. Also, when the jackal vocalisation was played during the playback phase, the same numbers of Koniks and Arabians were standing still (1LS: Koniks: *n* = 9, Arabians: *n* = 8, *p* > 0.05; 4LS: Koniks: *n* = 4, Arabians: *n* = 8, *p* > 0.05), standing alert (1LS: Koniks: *n* = 4, Arabians: *n* = 1, *p* > 0.05; 4LS: Koniks: *n* = 5, Arabians: *n* = 5, *p* > 0.05), walking (1LS: Koniks: *n* = 8, Arabians: *n* = 7, *p* > 0.05; 4LS: Koniks: *n* = 9, Arabians: *n* = 5, *p* > 0.05), and trotting or cantering (1LS: Koniks: *n* = 7, Arabians: *n* = 4, *p* > 0.05; 4LS: Koniks: *n* = 6, Arabians: *n* = 2, *p* > 0.05).

**Figure 2:**
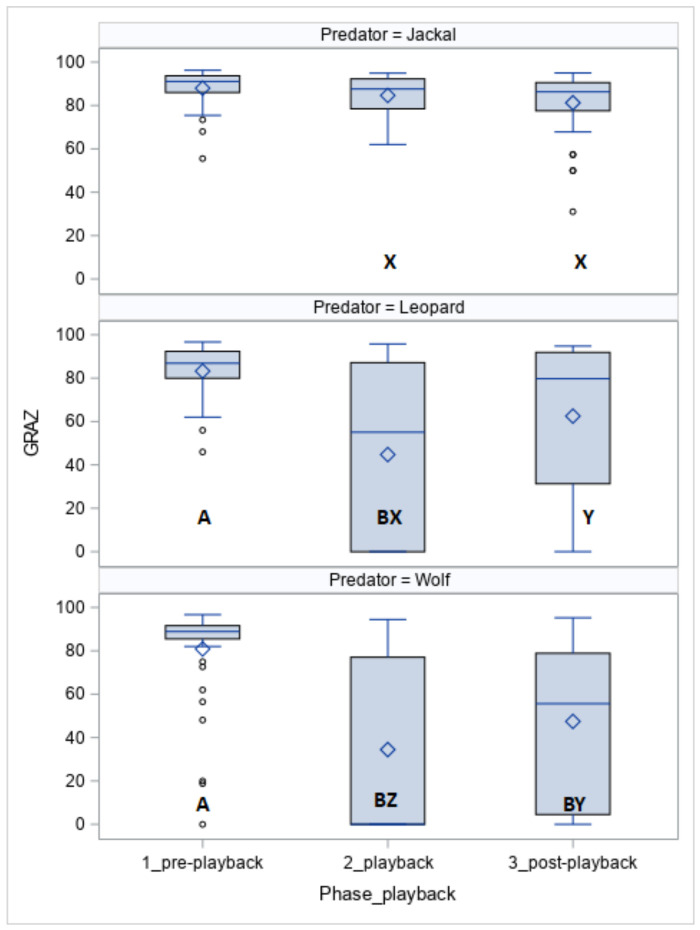
Duration of grazing (GRAZ; %) in horses exposed to the vocalisation of the jackal, leopard and wolf. Values with A, B letters differ significantly at *p* < 0.01 between phases 1–3 (pre-, playback and post-playback); values with X, Y, Z letters differs significantly at *p* < 0.01 between predators; values with no letter do not differ to other values.

The time spent on grazing (GRAZ, Figure 2) was affected by the phase (*p* < 0.05), the predator (*p* < 0.05), and the predator within the phase (*p* < 0.05). The horses grazed for less time when exposed to the leopard vocalisation (phase 1 vs. phase 2, *p* < 0.05) and wolf vocalisation (phase 1 vs. phase 2, *p* < 0.05 and phase 1 vs. phase 3, *p* < 0.05). Except for phase 1, the duration of grazing was longer for the jackal than for the leopard (phase 2, *p* < 0.05 and phase 3, *p* < 0.05) and wolf (phase 2, *p* < 0.05 and phase 3, *p* < 0.05).

More Koniks than Arabians grazed (1LS: *n* = 7, 70% vs. *n* = 2, 20%, *p* < 0.05 and 4LS: *n* = 8, 80% vs. 2, *p* < 0.05) when exposed to vocalisations of the leopard.

**Figure 3:**
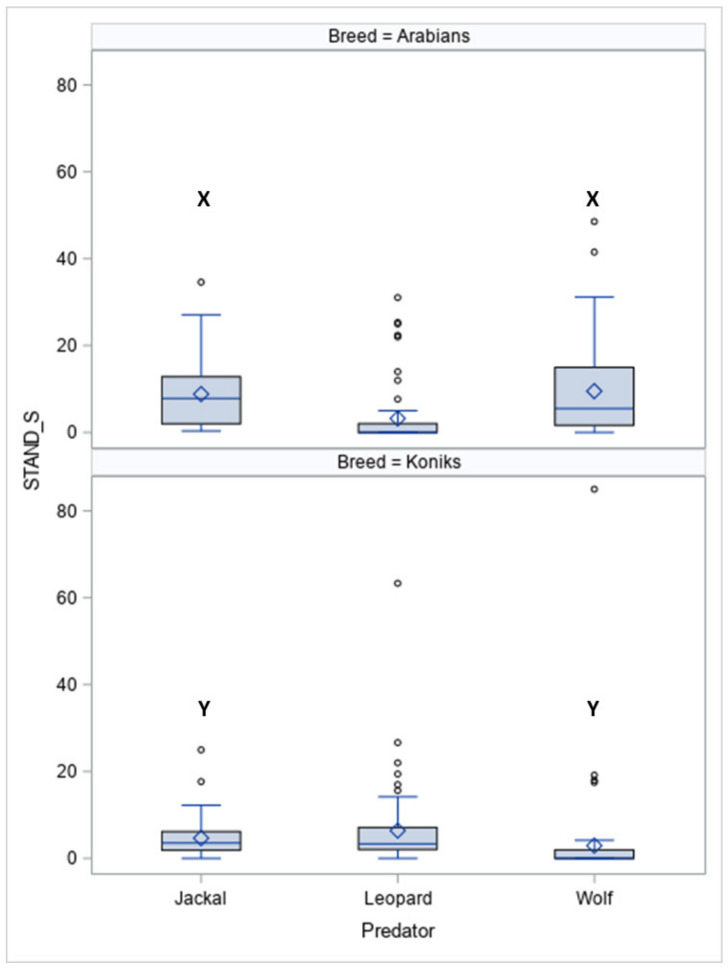
Duration of standing still (STAND-S, %) in Arabians and Koniks exposed to the vocalisation of the jackal, leopard and wolf. Values with X, Y letters differs significantly at *p* < 0.05 between breeds; values with no letter do not differ to other values.

The durations of standing still (STAND-S, Figure 3) differed between the breeds (*p* < 0.05) and between the predators within the breeds for jackal vocalisations (*p* < 0.05) and wolf vocalisations (*p* < 0.05).

In response to vocalisations of the leopard, more Koniks than Arabians stood still (1LS: 8, 80% vs. 1, 10%, *p* < 0.05 and 4LS: *n* = 7, 70% vs. *n* = 1, 10%, *p* < 0.05). In contrast, more Arabians than Koniks stood still when the wolf howls were played (1LS, *n* = 8, 80% vs. *n* = 0, 0%; *p* < 0.05 and 4LS: *n* = 7, 70% vs. *n* = 1, 10%, *p* < 0.05).

**Figure 4:**
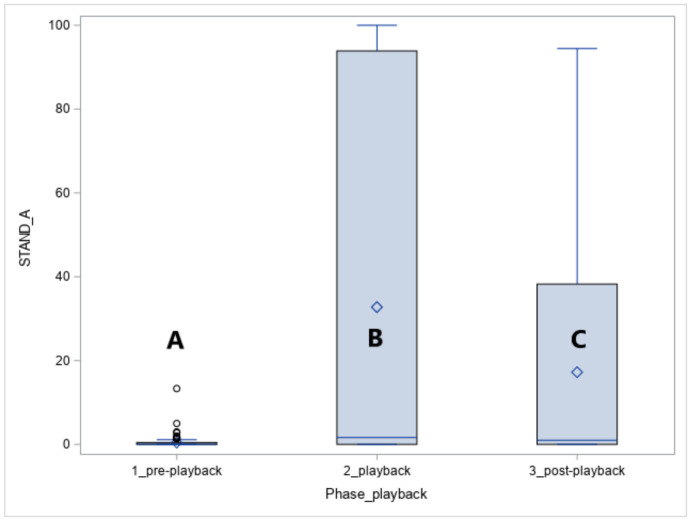
Duration of standing alert (STAND-A, %) in Arabians and Koniks exposed to the vocalisation of the jackal, leopard and wolf. Values with A, B and C letters differs significantly at *p* < 0.05 between breeds.

The vocalisation of the predators caused the alert standing posture in all horses (STAND-A, phase effect: *p* < 0.05, Figure 4). The alertness increased in response to the playback (phase 1 vs. phase 2, *p* < 0.05) and was also observed for the five minutes following the playback namely the post-playback phase (phase 1 vs. phase 3, *p* < 0.05).

In the playback phase, more Arabians than Koniks stood alert (1LS: *n* = 10, 100% vs. *n* = 6, 60%, *p* < 0.05) in response to leopard vocalisations whereas during the wolf vocalisations more Koniks than Arabians stood alert (both 1LS and 4LS: *n* = 10, 100% vs. *n* = 1, 10%, *p* < 0.05).

**Figure 5:**
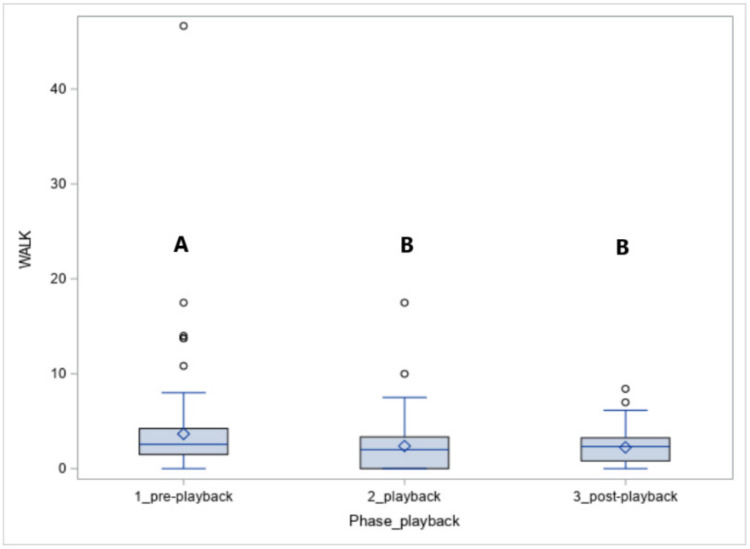
The number of steps in walk (WALK) in horses exposed to the vocalisation of predators. Values with A, B letters differ significantly at *p* < 0.01 between phases 1–3 (pre-, playback and post-playback).

The number of steps walked (WALK, Figure 5) was affected by the phase (*p* < 0.05). The horses walked more in phase 1 compared to phase 2 (*p* < 0.05) and phase 3 (*p* < 0.05).

During the exposure to leopard vocalisations, more Koniks than Arabians walked (1LS: 9, 90% vs. 1, 10%, *p* < 0.05 and 4LS: 6, 60% vs. 1, 10%, *p* < 0.05) but when the wolf howls were played, more Arabians than Koniks walked (1LS: *n* = 7, 70% vs. *n* = 1, 10%; *p* < 0.05 and 4LS: *n* = 6, 60% vs. *n* = 0, 0%, *p* < 0.05).

**Figure 6:**
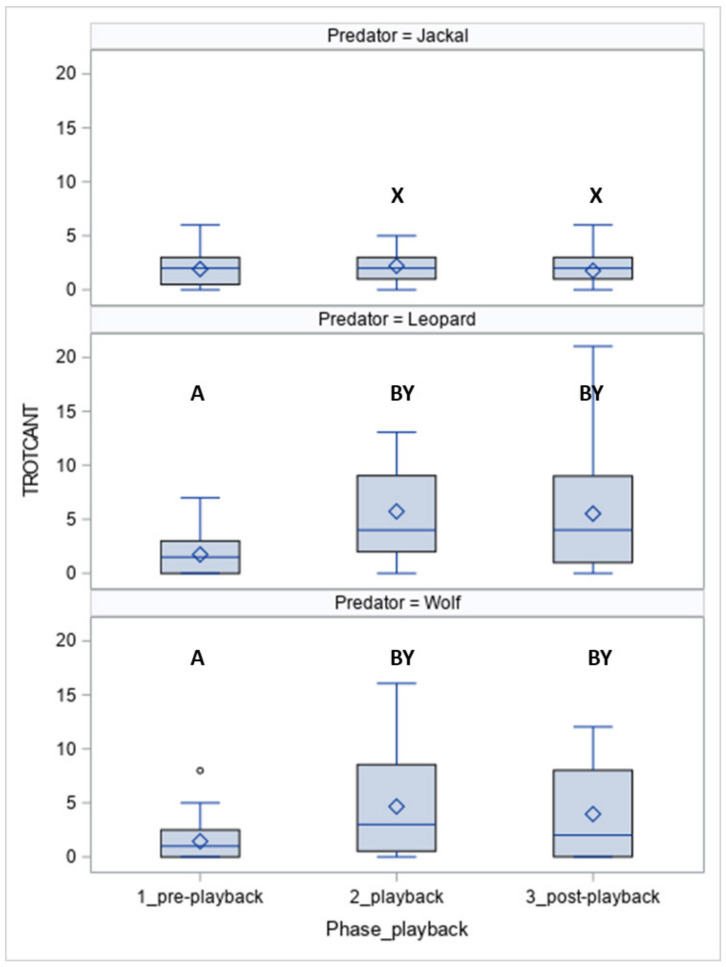
The number of steps in trot and canter (TROTCANT) in horses exposed to the vocalisation of predators. Values with A, B letters differ significantly at *p* < 0.01 between phases 1–3 (pre-, playback and post-playback); values with X, Y, Z letters differs significantly at *p* < 0.01 between predators. Values with no letter do not differ to other values.

The number of steps in more active gaits (TROTCANT, Figure 6) differed between predators (*p* < 0.05), phases (*p* < 0.05), and phases between and within predators (*p* < 0.05). When the leopard and wolf vocalisation was played, the TROTCANT was higher in phase 2 and phase 3 than in phase 1 (both *p* < 0.05). The horses made more steps in trot and canter when the leopard and wolf vocalisations were played compared to the jackal vocalisations (jackal vs. leopard, *p* < 0.05 and vs. wolf, *p* < 0.05) and in the post-playback phase (jackal vs. leopard, *p* < 0.05 and vs. wolf, *p* < 0.05).

More Arabians than Koniks trotted or cantered (1LS: 9, 90% vs. 3, 30%, *p* < 0.05 and 4LS: 10, 100% vs. 2, 20%, *p* < 0.05) during the leopard vocalisations. Again, wolf vocalisations stimulated more trotting or cantering in Koniks than in Arabians (1LS: *n* = 10, 100% vs. *n* = 3, 30%, χ^2^ = 10.77, *p* < 0.05 and 4LS: *n* = 10, 100% vs. *n* = 2, 20%, *p* < 0.05).

### 3.2. Distance to Reference Horse and Loudspeaker(s)

The distance to the reference horse (DIST-RH, Appendixes A and B) differed depending on the phase (*p* < 0.05), predator (*p* < 0.05), as well as on the interactions of the breed, predator and phase (*p* < 0.05). The Arabians responded with a decrease of the distance between individuals only when exposed to the leopard vocalisation (phase 1 vs. phase 2, *p* < 0.05 and phase 2 vs. phase 3, *p* < 0.05; Appendix A) while the Koniks grouped tightly when wolf howls were played (phase 1 vs. phase 2, *p* < 0.05 and phase 1 vs. phase 3, *p* < 0.05; Appendix B). Even if the majority of horses grouped around the reference animal during the playback of the leopard, certain individual Arabian horses tended to maintain bigger distance from the core of the herd in phase 3 (Appendix B).

The distance to the nearest loudspeaker (DIST-LS, Appendixes C and D) was affected by the phase (*p* < 0.05), the predator (*p* < 0.05), number of loudspeakers (*p* < 0.05) and interactions of the phase, predator and number of loudspeakers (*p* < 0.05). When the recording of the leopard was played back from 1LS, the Koniks cautiously approached the LS, but only after the leopard growl playback was stopped (phase 2 vs. phase 3, *p* < 0.05, Appendix C). In contrast, the Arabians approached the LS instantly while the leopard was played (phase 1 vs. phase 2, *p* < 0.05) and then withdrew in phase 3 (phase 2 vs. phase 3, *p* < 0.05, Appendix C). The spatial formation of the herd as assessed in the first 3 min was horizontally linear i.e., all animals approached the loudspeaker side-by-side. The Arabians also responded by approaching the loudspeakers when the leopard was played from 4LS (phase 1 vs. phase 2, phase 1 vs. phase 3, *p* < 0.05 and phase 2 vs. phase 3, *p* < 0.05, Appendix D); however, the linear formation was not observed in this instance. The reaction displayed by the Koniks when the wolf was played from four directions was opposite to that of the Arabians. The individuals attempted to bunch together and to maintain a larger distance from the source of the sound (phase 1 vs. phase 2, *p* < 0.05 and phase 1 vs. phase 3, *p* < 0.05, Appendix D).

## 4. Discussion

The results of our study confirmed that predator vocal cues alone triggered instantaneous changes in the grazing behaviour of horses, provoking alertness, engagement in higher gaits and grouping. The type of spatial formation (a bunch vs. a line) of the herd in response to the perceived presence of a predator appears to be linked to the breed, which is an important outcome. This demonstrates the probable strategy the equine herd employs during a potential risk of predation. The strong relationship between the breed and predator area of origin was also a highly notable outcome.

The way ungulate species respond to predators has been linked to the cover of their habitats [36] and the mode of hunting (chasing vs. stalking [20]). It has been proposed that in thick vegetation cover, ungulate species use auditory cues for the detection of predators, while, in more open spaces, the animals predominantly use visual cues [36]. This is the case for horses, which show an instinctive fear response to novel visual cues (e.g., [29,30,39]). Furthermore, quickly moving objects provoke impulsive escape responses, which may illustrate the strategy employed by horses in case of an attack from a predator, irrespective of the hunting mode (stalking vs. chasing).

The Asian origin of *Equus caballus*, which shared vast open habitat (steppes) primarily with the wolves [40,41] may explain an instinctive reaction of horses to wolf howls, which was the case for Koniks in the present study. Similarly the Eurasian habitats of aurochs shared with wolves during their domestication had been proposed as an explanation for the pronounced response of cattle to wolves as their natural predator [42]. The clear differences in behavioural response to predators between the two horse breeds are difficult to explain. Although the horses were not observed to be predated by the Arabian leopard [11], it can be very cautiously proposed that this breed, originating from the fertile crescent in the Middle East, may be more responsive to the growls of the leopards inhabiting the same area than to the howling of the wolf, absent in Africa. However, breed related differences in behaviour could be only incidental, so this explanation is highly speculative.

The predominant role of visual cues in assessment of threat by horses was confirmed by [24]. The combined predator cues (urine and sudden movement of a plastic bag) caused increased heart rate response and tendency to a delayed return to feeding compared with olfactory stimulus alone (increased sniffing and vigilance, decreased eating and more behavioural shifts). In the present study, the auditory stimulus alone was sufficient to provoke locomotor and alert reactions [34]. The horses interrupted their feeding, engaged in active (trot and canter) locomotive behaviour and presented increased attention to the environment. In the study of Ahmadinejad et al. [25] a lion’s roar provoked a significantly higher heart rate response than an olfactory cue. This might be related to the vocalisation of predators during hunting and is particularly true for canids that chase prey. Wolves growl during the harassment of prey and howl before [37] and during the chasing of prey, foxhounds bay when following a scent, African wild dogs yip or give mob-twitters and dholes (*Cuon alpinus*) use a variety of vocal signals during hunting [38]. The felids are known to vocalise before hunting, for example lions and cheetahs moan or hum in the pre-hunt phase [38]. Notwithstanding the breed, the horses mostly ignored the barking howls of the jackal. It may be supposed that vocalisations of a jackal, which is a smaller predator differing in frequency to that of a leopard or a wolf, and that this results in less interest in this particular predator. It has been proposed that, in mammals, the frequency of the voice serves as a reliable predictor of the body size [43]. Low-frequency sounds were found to be emitted by larger males of fallow deer (*Dama dama,* [44]), baboons (*Papio hamadryas*, [43]), sea elephants (*Mirounga leonina*, [45]) and lions (*Panthera leo*, [46]). Although such an ability was not confirmed in horses, they were able to recognise important information coded in vocal signals, e.g., the social status and familiarity of conspecific whinnying [47]. Thus, hypothetically, the information regarding the size of the predator, coded in vocalisation, could be instinctively deciphered by equines.

Individual and social responses to the vocal cues of each predator were seen in horses of both breeds; however, not all behaviours were equally displayed by Koniks and Arabians. Contrary to the expectation that the Arabians were more prone to alertness [48], they indeed stood still for longer periods than the Koniks when exposed to the vocalisations of jackals and wolves. The Koniks and Arabians displayed different behaviours in response to the vocalisation of predators. Firstly, the Koniks were more alert in response to the wolf vocalisations simulated by 4LS, while the Arabians were more responsive to the leopard growls. Secondly, the Koniks formed a cohesive group avoiding the purported predator, while the Arabians actively approached the loudspeakers in a linear formation. It may be hypothesized that the bunching may be a better strategy in response to group-hunting predators, while linear formation may be more effective in the case of a solitary hunting predator, but this is highly speculative. Increased vigilance, interruption of feeding (measured with the giving-up density), protection of the young, aggressive behaviours and the formation of anti-predator circles were also reported for large ungulates, such as bovids (e.g., bison (*Bison bison)*, [49]), muskoxen (*Ovibos moschatus*, [50]) and domestic cattle (*Bos taurus*, [42]); or cervids (e.g., mule deer (*Odocoileus hemionus*, [51], and elk *Cervus elaphus*, [52]).

While aggression towards dogs and grouping when frightened have been observed in daily practice with domestic horses, our study is the first to confirm that synchronized reactions and decreased distancing between individuals occur in this species as a social anti-predatory behaviour. Proximity to conspecifics in the case of a predator attack has many benefits, including a reduced risk of being caught [53]. The circular herd formation found in Koniks may be a better strategy than a linear formation to avoid wolf predation. The observed linear group formation in the Arabians could be only incidental; however, a variety of defensive formations in ungulates has been previously observed. For instance, elephants and bisons have all been observed to form defensive circles. Sheep and cows adopt different herd formations depending on their activities, such as grazing, resting, ruminating or in response to a presence of a predator [54]. The linear pro-active formation of the Arabians is in line with the proposition of Jarman and Jarman [55] that a herd taking the same velocity in response to stimuli is both the factor for and the result of a stable herd structure. In contrast, the consistency of the anti-predator response in domestic cattle was rather low. In four of eight events regarding presence of wolves in the pastures, the cattle grouping was characterized by no clear pattern and a high variation in distance while moving [20]). This is likely due to selection for tameness [21]. A more consistent response to taxidermy mounts of different predators in the robust “Old Norwegian” breed of sheep was confirmed [22]. For this reason, the robustness of the breed may be of importance when in the effectiveness of the social protective behaviour of horses is analysed.

Hypothetically, this result may explain the lack of wolf depredation of Koniks, the pony-sized horses living in semi-feral groups in forest sanctuaries as observed in Poland [56] in contrast to cases of ponies and foals of other breeds that are preyed upon in Spain, Slovenia, Italy, Portugal, Greece, Estonia and Croatia [6]). As young animals are the main target of the attack, the special herd formations and protective activity of the adults are an important strategy for the protection of the young. In bisons (*Bison bison*), the calf’s self-protection and social protection behaviour of running to the cow, running to the herd, running to the nearest bull and moving to the front and centre of the fleeing herd were observed to be successful strategies for protection [57]. The occurrence of groups too small for effective social antipredator behaviour, confines of enclosures preventing escape or the genetic background (the breed) may be potential factors for the wolf depredation more than solely the recent wolf population increase.

We are fully aware that our study has its limitation—namely, the lack of reference artificial control sound for each of the predators. This might be more explanative but also it would increase the number of test repetitions. It still remains that the fact of being alerted by unknown sounds may be indicative of horses being able to perceive any strange sounds as the endangerment that can come from the predators. We plan in the next studies to combine vocal and visual stimuli with respective control cues.

Also, the response of horses could be different if they were not enclosed in a restricted area. However, the observed behaviour was reported also by breeders of Hutzul horses who eye-witnessed the spatial organisation in free-pastured herds in reaction to wolves (personal communication to AGB). Despite the unlimited flying distance, the herd of adult Hutzul mares (with or without stallion) bunch, keeping the foals inside the herd formation. Such behaviour might explain higher efficiency in foal protection by bunched herds, as during the chase the foals could be an easier targets for wolves.

Our study is only a preliminary approach to the problem of the return of wolves to the countries where they were absent for many years. However, while horses are only incidentally predated upon, this low incidence still alarms horse owners and breeders and may potentially lead to the discontinuation of wolf protection in these countries. Present results suggest that keeping horses in groups may help the breeders to diminish the risk of predation, but more extensive studies are needed to explore horse-predators interaction. Due to unavailable scientific evidence of how wolves hunt horses, more field studies on horse-predator interactions are urgently needed. We believe that our preliminary results shed more light on the background of fearfulness in equines and are interesting for all studying horse-predator behaviour.

## 5. Conclusions

Our results confirmed the existence of behavioural response to vocalisations of predators and demonstrated that two breeds of horses applied different tactics in their social antipredator behaviour. Likely, horses are able to assess the size of a predator based on vocal cues. Adult horses were able to respond to potential predation as a group. Supposedly, this ability is one of the explanations for the least number of horses among all preyed farm animal species.

In view of a scarce reference to horse-predators studies, our preliminary observations on social protection behaviour is the first attempt in investigating this complex problem within the horse industry. As the management of both farm animals and large predators plays a role in the sustainable protection of both wildlife and human resources [58], further studies exploring predator-prey interactions in domestic animals are urgently needed.

## Figures and Tables

**Table 1:** Behaviours observed during 5-min pre-playback, playback and post-playback phases of the trial.

Behaviour (Abbreviation)	Definition (Measure)
Grazing (GRAZ)	Feeding with grass; duration (minutes)
Standing still (STAND-S)	Standing motionless, including resting position; duration (minutes)
Standing alert (STAND-A)	Standing with high head position, looking around, ears either fixed in the forward position or frequently changing position; duration (minutes)
Walking (WALK)	Slow four-beat gait; the number of steps
Trotting, cantering (TROTCANT)	Medium-speed two-beat gait; high-speed three-beat gait; the number of steps in both gaits

**Figure A1:**
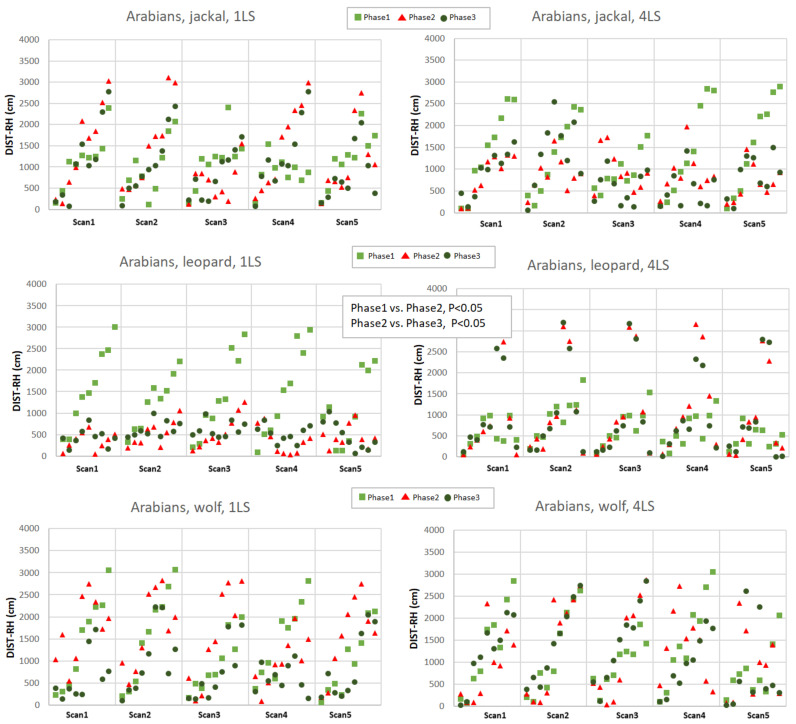
Distance to the reference horse (DIST-RH, cm) in Arabians in response to jackal’s, leopard’s and wolf’s vocalisation played from 1LS and 4LS in pre-, playback and post-playback phases. Each point represents the distance of individual horse from the RH. Phases are depicted with light green squares (phase 1, pre-playback), red triangles (phase 2, playback) and dark green circles (phase 3, post-playback). Only significant differences between phases were shown in the figure.

**Figure A2:**
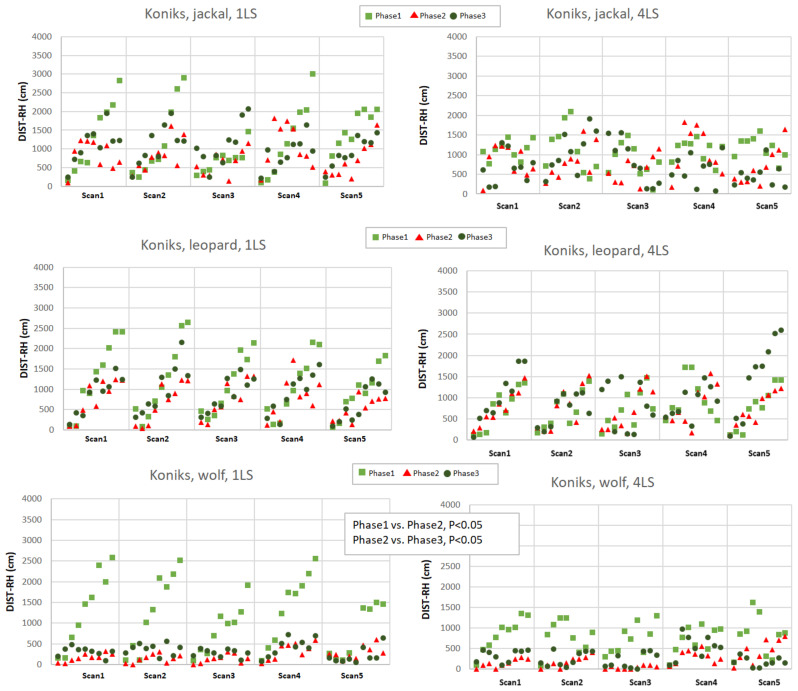
Distance to the reference horse (DIST-RH, cm) in Koniks in response to jackal’s, leopard’s and wolf’s vocalisation played from 1LS and 4LS in pre-, playback and post-playback phases. Each point represents the distance of individual horse from the RH. Phases are depicted with light green squares (phase 1, pre-playback), red triangles (phase 2, playback) and dark green circles (phase 3, post-playback). Only significant differences between phases were shown in the figure.

**Figure A3:**
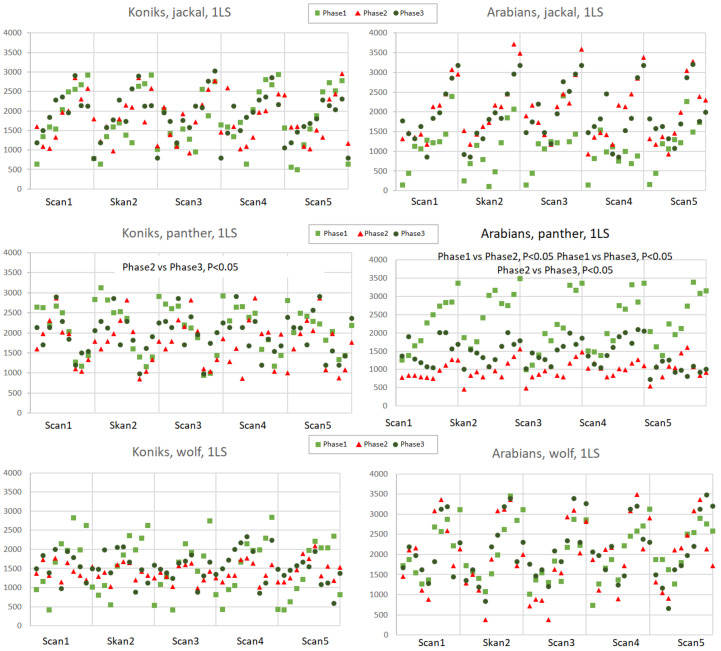
Distance to the nearest loudspeaker (DIST-LS, cm) in Koniks and Arabians in response to jackal’s, leopard’s and wolf’s vocalisation played from 1LS in pre-, playback and post-playback phases. Each point represents the distance of individual horse the nearest LS. Phases are depicted with light green squares (phase 1, pre-playback), red triangles (phase 2, playback) and dark green circles (phase 3, post-playback). Only significant differences between phases were shown in the figure.

**Figure A4:**
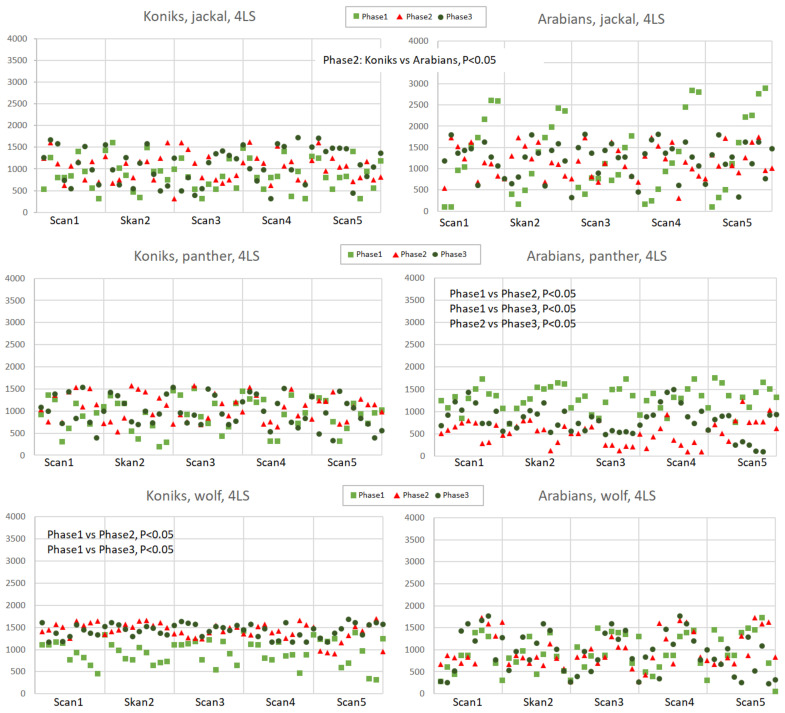
Distance to the nearest loudspeaker (DIST-LS, cm) in Koniks and Arabians in response to jackal’s, leopard’s and wolf’s vocalisation played from 4LS in pre-, playback and post-playback phases. Each point represents the distance of individual horse from the nearest LS. Phases are depicted with light green squares (phase 1, pre-playback), red triangles (phase 2, playback) and dark green circles (phase 3, post-playback). Only significant differences between breeds and phases were shown in the figure.
